# Which current chatbot is more competent in urological theoretical knowledge? A comparative analysis by the European board of urology in-service assessment

**DOI:** 10.1007/s00345-025-05499-3

**Published:** 2025-02-11

**Authors:** Mehmet Fatih Şahin, Çağrı Doğan, Erdem Can Topkaç, Serkan Şeramet, Furkan Batuhan Tuncer, Cenk Murat Yazıcı

**Affiliations:** Urology Department, Tekirdag Namık Kemal University, Süleymanpaşa, Tekirdağ, 59020 Turkey

**Keywords:** European board of urology, In-service assessment, Chatbot, GPT, Gemini, Copilot, Sonar huge, Claude

## Abstract

**Introduction:**

The European Board of Urology (EBU) In-Service Assessment (ISA) test evaluates urologists’ knowledge and interpretation. Artificial Intelligence (AI) chatbots are being used widely by physicians for theoretical information. This research compares five existing chatbots’ test performances and questions’ knowledge and interpretation.

**Materials and methods:**

GPT-4o, Copilot Pro, Gemini Advanced, Claude 3.5, and Sonar Huge chatbots solved 596 questions in 6 exams between 2017 and 2022. The questions were divided into two categories: questions that measure knowledge and require data interpretation. The chatbots’ exam performances were compared.

**Results:**

Overall, all chatbots except Claude 3.5 passed the examinations with a percentage of 60% overall score. Copilot Pro scored best, and Claude 3.5’s score difference was significant (71.6% vs. 56.2%, p = **0.001**). When a total of 444 knowledge and 152 analysis questions were compared, Copilot Pro offered the greatest percentage of information, whereas Claude 3.5 provided the least (72.1% vs. 57.4%, p = **0.001**). This was also true for analytical skills (70.4% vs. 52.6%, p = **0.019**).

**Conclusions:**

Four out of five chatbots passed the exams, achieving scores exceeding 60%, while only one did not pass the EBU examination. Copilot Pro performed best in EBU ISA examinations, whereas Claude 3.5 performed worst. Chatbots scored worse on analysis than knowledge questions. Thus, although existing chatbots are successful in terms of theoretical knowledge, their competence in analyzing the questions is questionable.

## Introduction

Current chatbots are open-source, language model-based artificial intelligence (AI) systems that are designed to comprehend and produce human-like answers to the questions asked [1]. Today, patients frequently use chatbots to learn about their medical conditions. Healthcare professionals and clinicians can also use chatbots to learn about medical issues quickly. These chatbots have the potential to receive information from many sources. However, in daily practice, the ideas put forward by interpreting patient evaluation, anamnesis, physical examination, laboratory findings, and imaging methods are the characteristics that make a clinician successful [2]. In addition to pure knowledge, interpreting the data and obtaining some results through analysis should be a characteristic specific to humans and an ability targeted by AI. Chatbots potential uses include diagnosis, outcome prediction, therapy planning, and evaluation of surgical methods in urology. AI may contribute to urology education and training. AI facilitates rapid access to contemporary research in urological surgery, streamlining the acquisition of knowledge that formerly required years to get. To test this, an appropriate method would be to evaluate the board exams, an evaluation with high knowledge measurement features that are valid throughout Europe and the World.

Recently, many studies have assessed ChatGPT’s overall capacity to pass medical examinations [3]. The success of current chatbots in questions that measure knowledge is increasing daily. However, which of the many chatbots available is the most successful? Do these chatbots also have the ability to synthesize information and interpret it correctly? This research aimed to assess the overall capability of several chatbots in responding to the European Board of Urology In-Service Assessment (EBU ISA) questions, which closely resemble the official questions of the EBU written test. Also, it aimed to evaluate and compare current chatbot performances in answering EBU exam questions, which is an exam that measures theoretical knowledge.

## Materials and methods

The Fellow of the European Board of Urology (FEBU) designation is conferred to candidates who complete the EBU exams, signifying a superior degree of expertise in urology. The title may only be acquired after many academic and practical training years. The European Association of Urology (EAU) annually conducts a two-step (EBU) examination, including written and oral components. To specialize in urology per the EBU standards, candidates must complete a written test (Part 1) and an oral examination (Part 2). The EBU provides applicants with the In-Service Assessment (ISA), a 100-question test with four multiple-choice options, to prepare for the Part 1 test. Before qualifying for the FEBU designation, each applicant is allowed to prepare and evaluate their knowledge via an ISA evaluation [4]. This examination includes questions that assess extensive factual knowledge according to current EAU guidelines, with a limited number of questions requiring analysis and interpretation of this material to obtain the correct response.

On September 15, 2024, the Urology Department at Tekirdag Namik Kemal University (Tekirdag, Turkey) performed research that did not include procedures on live organisms or human data, hence negating the need for ethics committee permission. No patient data were used since this study is unrelated to clinical research. Personal browser data were deleted before the searches were conducted to eliminate bias. All ISA questions were solely and discreetly supplied by the Executive Committee of the EBU for this research, precluding any potential of previous chatbot training. We examined three distinct test examinations: Exam 1 (2017–2018), Exam 2 (2019–2020), and Exam 3 (2021–2022). One examination had approximately 200 multiple-choice questions covering several subtopics, such as andrology, functional, lithiasis, oncology, pediatrics, surgery, transplantation, and trauma. One was formally designated as the correct answer among the four provided responses.

We individually posed all the questions to the latest and licensed (paid) versions of five different chatbots. We asked them to answer correctly according to the EAU guidelines for the exam year: GPT-4o (https://openai.com/gpt-4/) (May 2024 version), Claude-3.5 Sonnet (https://claude.ai/) (June 20, 2024 version), Copilot Pro (https://copilot.microsoft.com/) (January 2024 version), Gemini Advanced (https://gemini.google.com/) (2024 version), and Sonar Huge (August 2024 version), which is an advanced model trained by Perplexity. The original order of searches was maintained, and each query was processed on a separate web page to ensure separation and improve the analytical procedure. Distinct accounts were established for interaction with each AI chatbot to ensure noticeable differences. Before initiating the searches, all browser data were entirely erased.

All questions were analyzed one by one by two experienced urologists with FEBU titles (M.F.Ş. and Ç.D.) and were divided into two: those that measured knowledge and those that required interpretation. In case of a discrepancy, a consensus was reached by another experienced urologist with a FEBU title (C.M.Y.). Detailed subgroup analyses of all questions were analyzed individually and comparatively. Overall percentages of 60% or more were deemed as passing. Statistical analysis was conducted using SPSS Statistics (v29.0; Armonk, NY, USA). Categorical variables are expressed as n (%). Data were examined using Pearson’s chi-square test. Statistical significance was established at *p* < 0.05.

## Results

**Fig. 1:**
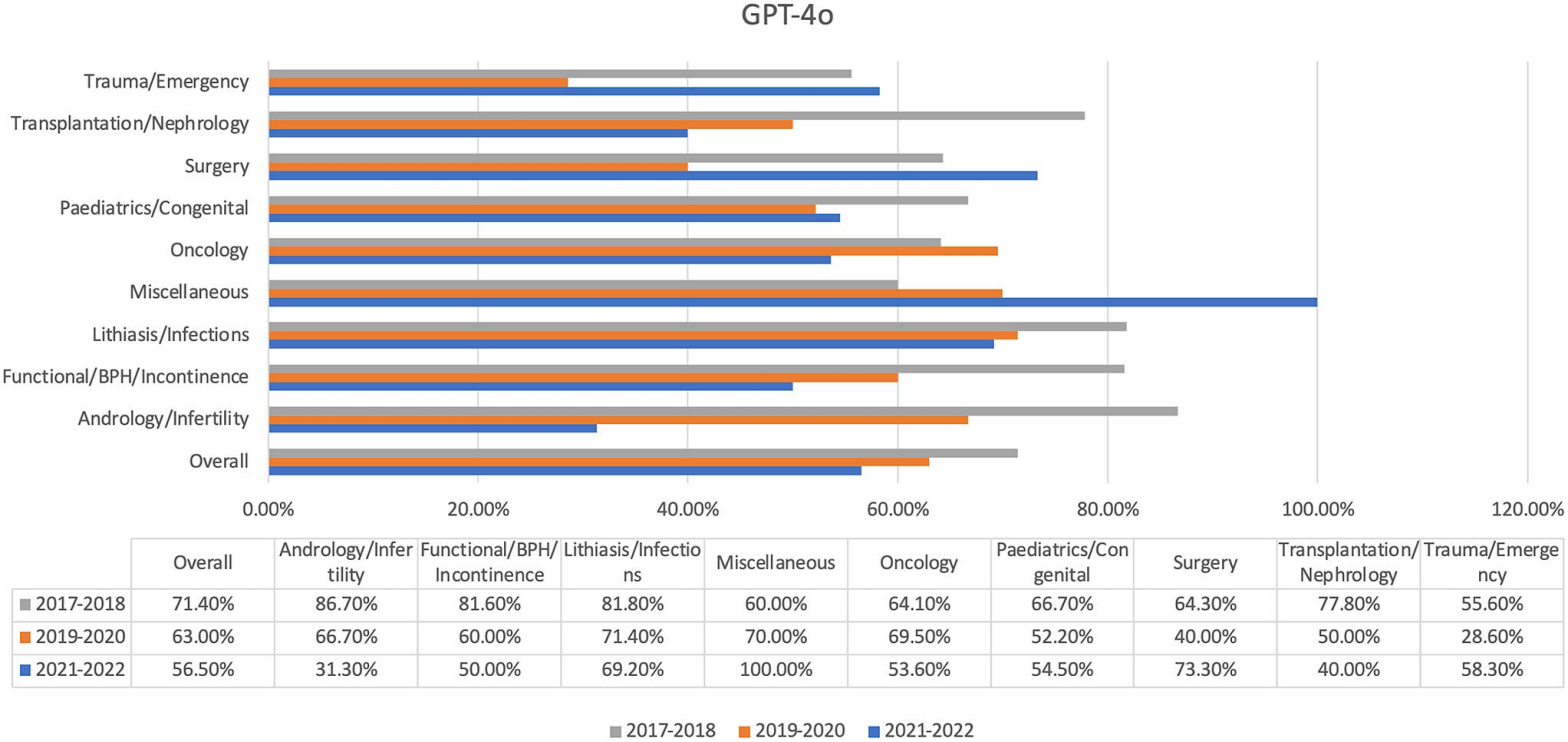
GPT-4o’s results

A total of 596 questions were analyzed. Distinct performance trends were observed in five chatbots that were evaluated. GPT-4o had a success rate of 65.8% and passed all three exams. The highest result was in Exam 1 (71.4%), while the lowest was in Exam 3 (56.5%) (Fig. 1). In terms of specific topic performance, GPT-4o showed consistent advantages in lithiasis/infections (73.7%) and miscellaneous (73.0%), while it had difficulties in trauma/emergency (50.0%) and transplantation/nephrology (61.1%).

**Fig. 2:**
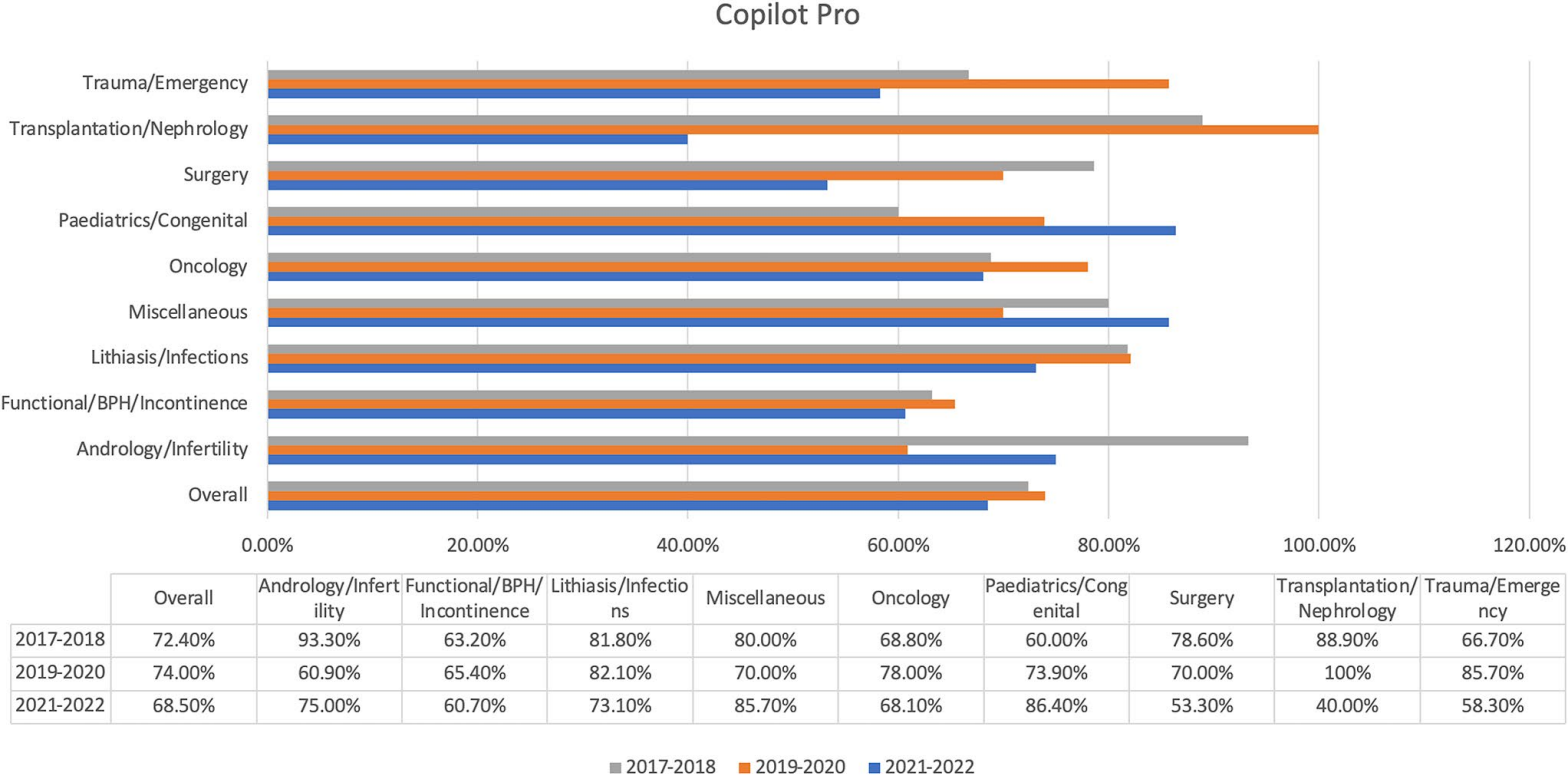
Copilot Pro’ results

Copilot Pro was undeniably the best-performing chatbot, as it was the one that succeeded the most overall at the impressive rate of 71.6% (Fig. 2). The bot made an extraordinary achievement in transplantation/nephrology, as it had the most correct answers (77.8%) and was the only bot with a high score of 100% in the transplantation section of Exam 2. In addition, Copilot Pro was also the leader in pediatrics/congenital (75.0%) and andrology/infertility (72.7%).

**Fig. 3:**
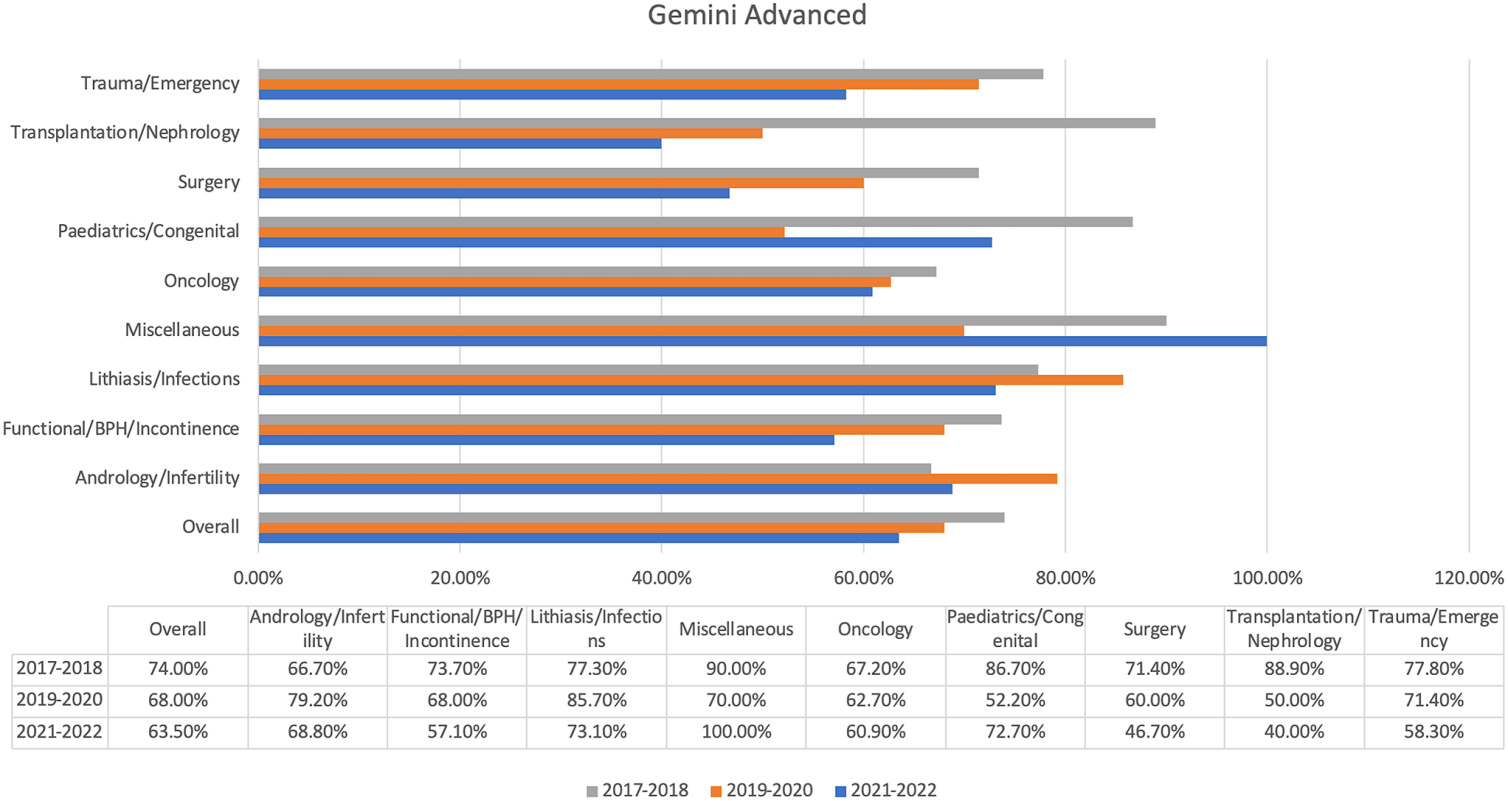
Gemini Advanced’ results

Gemini Advanced displayed an overall percentage of 68.5%, the mark after Copilot Pro and the second-best (Fig. 3). The chatbot was the best in miscellaneous (81.1%) and achieved the highest score of all chatbots (Fig. 3). However, its trauma/emergency and surgery subtopics were only moderately successful, as the chatbot achieved success rates of 67.9% and 59.0%, respectively.

**Fig. 4:**
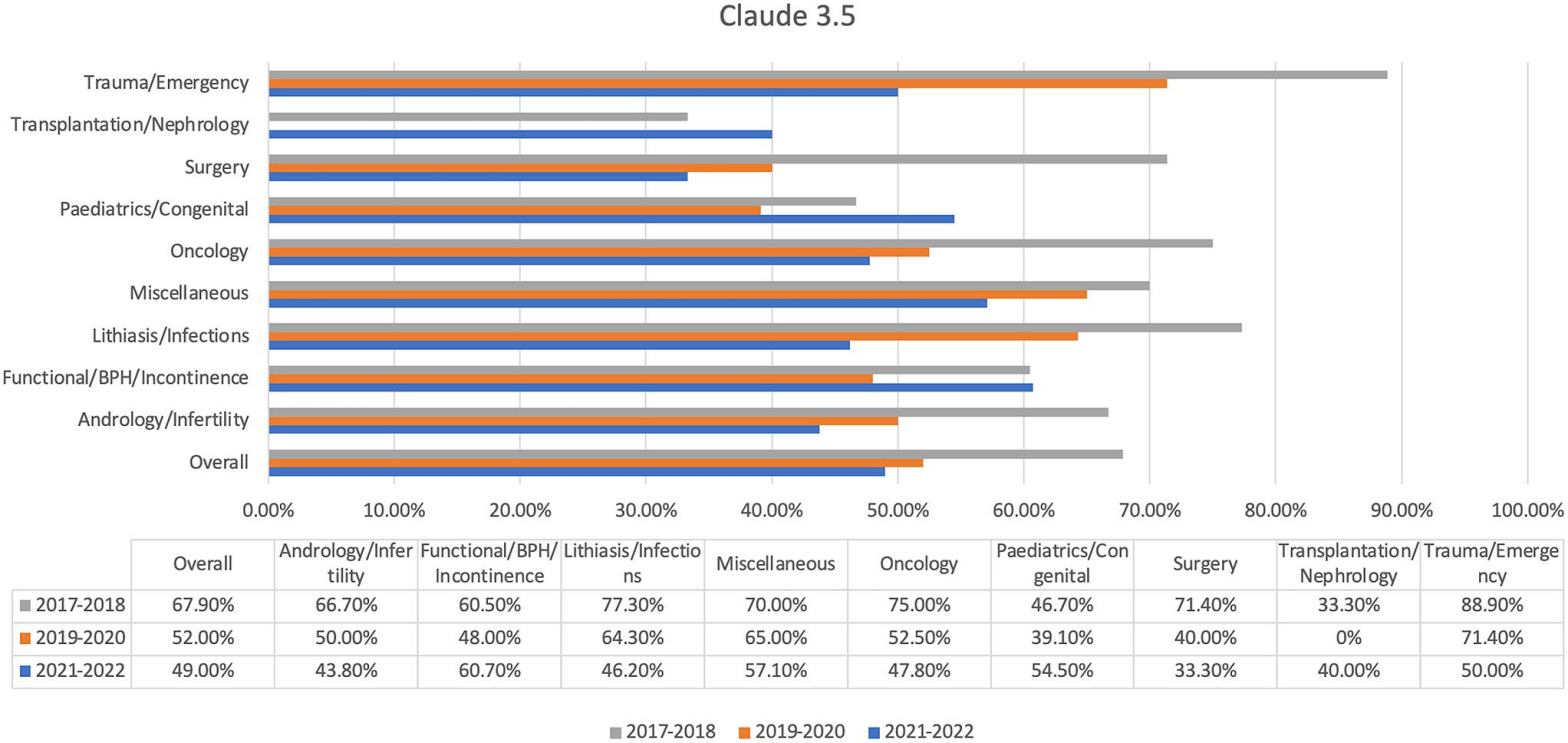
Claude 3.5’s results

Claude 3.5 was the least successful chatbot, with a pass rate of 56.2% (Fig. 4). It could not pass Exam 3 and had noticeably weak spots in transplantation/nephrology (27.8%) and pediatrics/congenital (46.7%). The chatbot had little success in functional/BPH/incontinence (57.1%), but it was much weaker in the sections other chatbots got.

**Fig. 5:**
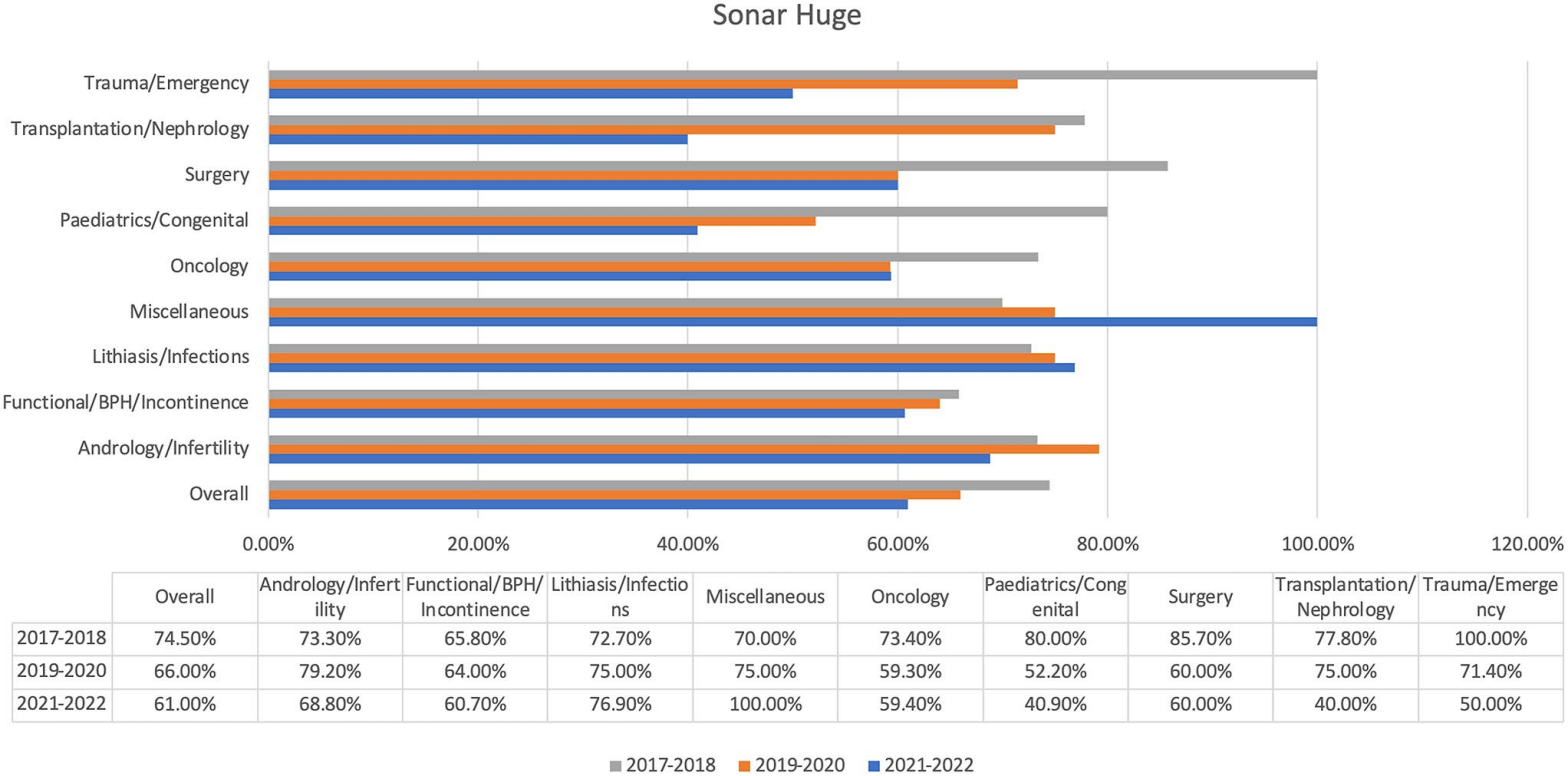
Sonar Huge’s results

Sonar Huge’s overall score was 67.1%, between Gemini Advanced and GPT-4o (Fig. 5). Additionally, it performed excellently in the miscellaneous (78.4%) and trauma/emergency (71.4%) sections, tying with Gemini Advanced in some sections. The weakest performance of Sonar Huge was in transplantation/nephrology (66.7%).

**Fig. 6:**
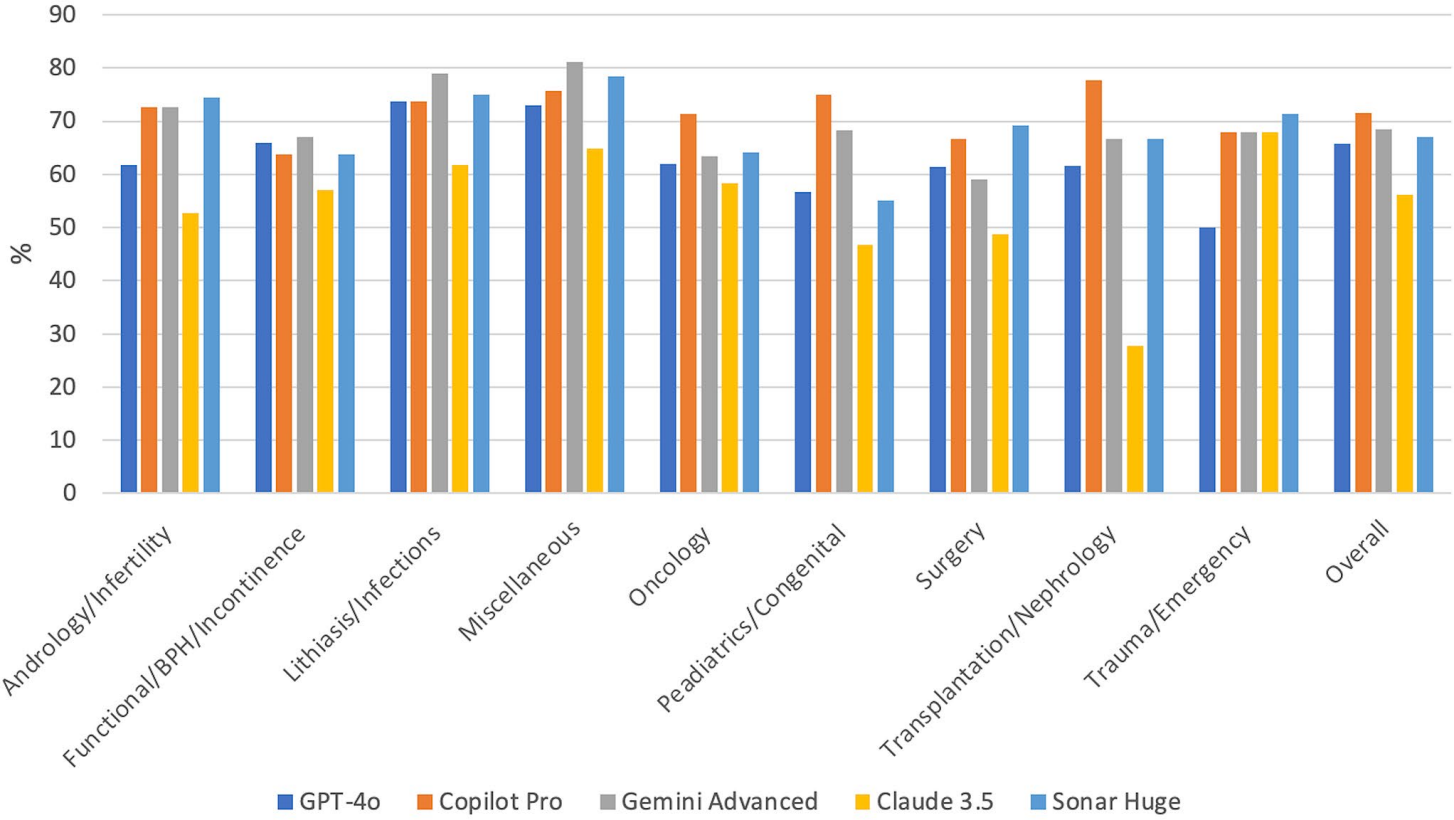
Overall results of the five chatbots with subtopics

In comparing five different chatbots, Copilot Pro was the most successful chatbot in the paediatrics/congenital category. At the same time, Claude 3.5 had the lowest score with a statistically significant difference between them (75.0% vs. 46.7%, p = **0.013**). In the transplantation/nephrology subtopic, Claude was the weakest, and the Copilot Pro was the strongest (77.8% vs. 27.8%, p = **0.030**). In terms of overall success, all chatbots, except Claude 3.5, were accepted as successful in the exams. Copilot Pro had the highest score among all, and Claude 3.5’s score difference was statistically significant (71.6% vs. 56.2%, p = **0.001**) (Table 1) (Fig. 6).

**Table 1:** Comparison of 5 different chatbot’s exam scores with subtopic distribution

	GPT-4o	Copilot Pro	Gemini Advanced	Claude 3.5	Sonar Huge	*p*
Andrology/Infertility (*n, %*)						
Incorrect	21 (38.2%)	15 (27.3%)	15 (27.3%)	26 (47.3%)	14 (25.5%)	0.069
Correct	34 (61.8%)	40 (72.7%)	40 (72.7%)	29 (52.7%)	41 (74.5%)	
Functional/BPH/						
Incontinence (n, %)						
Incorrect	31 (34.1%)	33 (36.3%)	30 (33.0%)	39 (42.9%)	33 (36.3%)	0.689
Correct	60 (65.9%)	58 (63.7%)	61 (67.0%)	52 (57.1%)	58 (63.7%)	
Lithiasis/Infections (n, %)						
Incorrect	20 (26.3%)	20 (26.3%)	16 (21.1%)	29 (38.2%)	19 (25.0%)	0.105
Correct	56 (73.7%)	56 (73.7%)	60 (78.9%)	47 (61.8%)	57 (75.0%)	
Miscellaneous (n, %)						
Incorrect	10 (27.0%)	9 (24.3%)	7 (18.9%)	13 (35.1%)	8 (21.6%)	0.593
Correct	27 (73.0%)	28 (75.7%)	30 (81.1%)	24 (64.9%)	29 (78.4%)	
Oncology (n, %)						
Incorrect	73 (38.0%)	55 (28.6%)	70 (36.5%)	80 (41.7%)	69 (35.9%)	0.112
Correct	119 (62.0%)	137 (71.4%)	122 (63.5%)	112 (58.3%)	123 (64.1%)	
Peadiatrics/Congenital (n, %)						
Incorrect	26 (43.3%)	15 (25.0%)	19 (31.7%)	32 (53.3%)	27 (45.0%)	0.013
Correct	34 (56.7%)	45 (75.0%)	41 (68.3%)	28 (46.7%)	33 (55.0%)	
Surgery (n, %)						
Incorrect	15 (38.5%)	13 (33.3%)	16 (41.0%)	20 (51.3%)	12 (30.8%)	0.403
Correct	24 (61.5%)	26 (66.7%)	23 (59.0%)	19 (48.7%)	27 (69.2%)	
Transplantation/						
Nephrology (n, %)						
Incorrect	7 (38.9%)	4 (22.2%)	6 (33.3%)	13 (72.2%)	6 (33.3%)	0.03
Correct	11 (61.1%)	14 (77.8%)	12 (66.7%)	5 (27.8%)	12 (66.7%)	
Trauma/Emergency (n, %)						
Incorrect	14 (50.0%)	9 (32.1%)	9 (32.1%)	9 (32.1%)	8 (28.6%)	0.495
Correct	14 (50.0%)	19 (67.9%)	19 (67.9%)	19 (67.9%)	20 (71.4%)	
Overall (n, %)						
Incorrect	204 (34.2%)	169 (28.4%)	188 (31.5%)	261 (43.8%)	196 (32.9%)	0.001
Correct	392 (65.8%)	427 (71.6%)	408 (68.5%)	335 (56.2%)	400 (67.1%)	

When the exam questions were categorized, 444 (74.5%) questions measured knowledge, and 152 (25.5%) questions were based on interpretation and analysis. The distribution of these questions according to subtopics is given in Table 2.

**Table 2:** Distribution of the knowledge and analysis questions among subtopics

	Knowledge	Analysis	Total
Andrology/Infertility	50 (90.9%)	5 (9.1%)	55
Functional/BPH/Incontinence	63 (69.2%)	28 (30.8%)	91
Lithiasis/Infections	56 (73.7%)	20 (26.3%)	76
Miscellaneous	30 (81.1%)	7 (18.9%)	37
Oncology	144 (75.0%)	48 (25.0%)	192
Peadiatrics/Congenital	43 (71.7%)	17 (28.3%)	60
Surgery	23 (59.0%)	16 (41.0%)	39
Transplantation/Nephrology	17 (94.4%)	1 (5.6%)	18
Trauma/Emergency	18 (64.3%)	10 (35.7%)	28
Overall	444 (74.5%)	152 (25.5%)	596

When comparing the types of questions, the chatbot that provided the most correct information was again Copilot Pro, while the chatbot that provided the least information was Claude 3.5. (72.1% vs. 57.4%, p = **0.001**). This was also the same when comparing analysis abilities (70.4% vs. 52.6%, *p = ****0.019***) (Table 3).

**Table 3:** Comparison of 5 different chatbot’s exam scores in terms of knowledge and analysis

	GPT-4o	Copilot Pro	Gemini Advanced	Claude 3.5	Sonar Huge	*p*
Knowledge (*n, %*)						
Incorrect	164 (36.9%)	124 (27.9%)	127 (28.6%)	189 (42.6%)	136 (30.6%)	0.001
Correct	280 (63.1%)	320 (72.1%)	317 (71.4%)	255 (57.4%)	308 (69.4%)	
Analysis (n, %)						
Incorrect	53 (34.9%)	45 (29.6%)	52 (34.2%)	72 (47.4%)	60 (39.5%)	0.019
Correct	99 (65.1%)	107 (70.4%)	100 (65.8%)	80 (52.6%)	92 (60.5%)	

## Discussion

Our research indicates that most of the latest chatbot versions, such as Copilot, Gemini, Sonar Huge, and ChatGPT, can successfully complete EBU-ISA examinations. Although studies compare different versions of ChatGPT [1], this is the first study to compare different chatbot performances of EBU-ISA exams. Older versions of ChatGPT have been shown to pass various medical exams, but they need improvements for urology board exams [5–7]. Nevertheless, the current research does not simply determine the extent to which these chatbot models can pass the EBU-ISA examinations. However, it also investigates those models’ relative strengths and weaknesses in answering different question types, like multiple-choice, clinical scenarios, and case-based reasoning. By identifying these patterns, we strive to determine if particular chatbots have a characteristic advantage in dealing with complex, high-stakes medical knowledge domains and analysis ability typically used for urology certification.

AI chatbots provide significant potential to enhance medical education by aiding in retaining factual information and developing practical skills. They have substantially improved medical students’ examination performance when used as an adjunct instructional resource. Students using the Chatprogress chatbot performed superior in pulmonology and general medical examinations compared to their peers who did not engage with the chatbot [8]. AI chatbots have effectively served as instructional assistance tools in public health training, surpassing medical students in addressing intricate medical inquiries about vaccination [9]. Medical students have a favorable disposition toward using AI and chatbots in their curriculum, acknowledging the prospective advantages and voicing apprehensions about data privacy and surveillance [10]. Medical education augmented by chatbots is anticipated to be a rapidly expanding domain, contingent upon enhancements in the accuracy and adherence to guidelines of the suggestions provided by these chatbots. Nonetheless, the need for a dependable, educational tool demands regular accuracy, which many chatbots have yet to provide effectively.

ChatGPT-4 exhibited exceptional performance on practice questions for the United States Medical Licensing Examination (USMLE), accurately responding to a significant percentage of questions across several test stages (Step 1, Step 2CK, and Step 3) [11]. Conversely, an older version of ChatGPT demonstrated modest efficacy, accurately responding to around 50% of the ophthalmology board certification practice inquiries [12]. Kollitsch et al. also found that, ChatGPT-4 and Bing AI consistently attained scores above the passing criterion for the EBU examination throughout all four testing cycles, however ChatGPT-3.5 did not [13]. In another study by May et al., it was found that ChatGPT-3.5, ChatGPT-4, and Bing AI display inadequate urological knowledge and are insufficiently proficient for instructional applications [14]. The fact that the latest version, GPT-4o, was successful on average in the FEBU exams shows us that new AI chatbots with more knowledge can be developed with technological developments.

When we performed the subtopic analysis, taking the average of all bots, we found that the lowest scores were for transplantation, at 60.0%, similar to the study by Schoh et al. [1], and pediatrics, at 60.3%. Regarding the subtopic of transplantation/nephrology, a potential explanation for the inadequate performance may be the limited number of questions, totaling just 18 in 3 examinations. Furthermore, chatbots have an impressive repository of factual information but struggle with contextual inquiries, particularly those about clinical, surgical, or anatomical subjects. Nevertheless, inquiries about transplantation often evaluate the positional connections among various organs, which may serve as an additional rationale for the poor performance in this area. The subtopic with the highest average was miscellaneous, with a success average of 74.6%, followed by lithiasis/infections (72.6%) and andrology (66.9%). This can be explained by the fact that in the miscellaneous section, queries are usually made from an extensive range of questions, and chatbots provide information flow from a wide range of areas via AI. Urinary lithiasis and urinary tract infections are some of the most common topics of interest to urology. Scientific studies are frequently conducted on these topics, and patients frequently use the internet because they are frequently experienced. Therefore, AI chatbots may have information about these topics and answer questions correctly at a high rate. Similarly, andrology can have a high accuracy rate due to the increasing frequency of studies and a popular topic with a pool of information.

One aspect that makes our study unique is that the questions are compared as questions that measure knowledge and require the analysis of information to reach the correct conclusion. Clinicians can use chatbots to get quick answers about information. Asking a question to a chatbot may often seem more practical than opening the relevant guideline and reading the desired information. However, as a result of our study, we should not trust this answer, and we have to consider that we may get a wrong answer at a rate of 33.3%. For these reasons, we should obtain knowledge through reading, learning, and personal development. Analysis questions are generally selected from clinical cases and similar to cases that urologists may encounter in clinics and real life. More than having pure knowledge is needed in these types of questions and cases; it is necessary to analyze this information with additional data and reach a conclusion. When we examine whether chatbots, the primary starting point of the study, possess the ability to analyze and interpret data, we find that the error rate of 37.1% is relatively high. Considering that correct diagnosis is very important on the way to patient health and correct treatment, it can be concluded from these results that current chatbots are far from replacing urologists in patient evaluation. Even the most current version of GPT, which is the most frequently used chatbot, has an accuracy rate of 65.1%, and this shows us that chatbots still have a long way to go in healthcare system. With the integration of current EAU guidelines, higher question answering and success rates, more accurate patient evaluation and clinical assessment will be possible.

Nonetheless, our analysis has many limitations: First, the question pool comprises ISA test examinations since the original written EBU exam questions were unavailable. Secondly, since the real results of the analyzed exams were not available, the chatbot answers could not be compared with the actual participants. Thirdly, only five popular chatbots were selected for comparison, and no comments can be made about others available. Lastly, although it is the most analogous preparatory examination of the FEBU, the 100 multiple-choice questions from the EBU-ISA may not thoroughly assess the knowledge of the five chatbots in urology and its subdisciplines.

## Conclusions

Our study revealed that 4 out of 5 analyzed chatbots successfully passed the 60% score in EBU-ISA exams. Based on answering EBU ISA questions, this study determined that the most successful chatbot was Copilot Pro, while the least successful was Claude 3. Chatbots scored lower scores in analysis questions than in questions that tested knowledge. Chatbots such as ChatGPT will evolve swiftly, significantly improving their performance on theoretical multiple-choice urological questions. Significant disparities in chatbot performance are determined throughout urological subfields. Specialized training of these chatbots in medicine is essential before confidently endorsing these instruments for acquiring urological knowledge and their efficient integration into medical education.

## Data Availability

No datasets were generated or analysed during the current study.

## Declarations

### R scientific responsibility statement

The authors declare that they are responsible for the article’s scientific content including study design, data collection, analysis and interpretation, writing, some of the main line, or all of the preparation and scientific review of the contents and approval of the final version of the article.
